# Profil épidémiologique de l'infection à VIH au cours d'une campagne de sensibilisation à Yaoundé au Cameroun

**DOI:** 10.11604/pamj.2013.15.119.2968

**Published:** 2013-08-05

**Authors:** Francois-Xavier Mbopi-Keou, Georges Nguefack-Tsague, Ginette Claude Mireille Kalla, Lade Viche, Michel Noubom

**Affiliations:** 1 Faculty of Medicine & Biomedical Sciences, University of Yaounde I, Yaounde-Cameroon; 2 National Public Health Laboratory, Ministry of Public Health, Yaounde, Cameroon; 3 University Teaching Hospital, Yaounde-Cameroon

**Keywords:** Dépistage du VIH, prévalence, profil épidémiologique, campagne de sensibilisation, HIV screening, prevalence, epidemiological profile, awareness campaign

## Abstract

**Introduction:**

L'objectif de ce travail était de déterminer le profil épidémiologique de l'infection à VIH/SIDA au cours d'une campagne de sensibilisation à Yaoundé au Cameroun.

**Méthodes:**

Après avoir obtenu le consentement éclairé des participants, le dépistage de l'infection par le VIH a été effectué selon l'algorithme de dépistage en série de l'Organisation Mondiale de la Santé (OMS). En outre, un questionnaire socio-comportemental a été proposé à chaque participant.

**Résultats:**

Au total, 911 personnes ont été dépistées. La prévalence de l'infection par le VIH était de 2.6%. Elle était 3 fois plus élevée parmi les femmes (RC = 3.22, IC (1.26 - 8.18). Les prévalences les plus élevées ont été observées chez les personnes de plus de 45 ans (p = 0.01), les personnes sans emploi (p < 0.001) et les veufs/veuves (p = 0.02). Plus de 80% des participants trouvaient le test de dépistage nécessaire et 76,2% l'avaient déjà effectué au moins une fois auparavant. Il s'agissait principalement de femmes (p = 0.02), d'étudiants (p < 0.001) et des personnes âgées de 25 à 34 ans (p < 0.001). Les personnes séropositives avaient moins tendance à retirer leur résultat (p = 0.01).

**Conclusion:**

Il apparait urgent d'intensifier les campagnes de dépistage de l'infection par le VIH en ciblant davantage des groupes particuliers tels que les élèves, les personnes âgées et les veufs/veuves, tout en recherchant les facteurs pouvant favoriser la propagation de l'infection dans ces groupes.

## Introduction

L'infection à VIH représente de part le monde un problème majeur de santé publique [1]. Au Cameroun, la prévalence de l'infection par le VIH a connue une progression constante [2–6], même si des récentes enquêtes ont démontré un ralentissement de la courbe d'évolution de l'épidémie [4, 7].

L'ignorance du statut sérologique constitue un facteur important de propagation de la maladie. Sa connaissance participe en effet de la première étape pour l'accès aux soins; une prise en charge précoce permettant de retarder le développement de la maladie [8]. Selon l'Organisation Mondiale de la Santé (OMS), dans les régions les plus affectées du monde, moins d'une personne sur dix infectées par le VIH connaît son statut sérologique [9]. En outre, la couverture mondiale des services de conseil et de dépistage du VIH demeure insuffisante, particulièrement en Afrique Subsaharienne [10]. C'est ainsi que l'approche mobile du dépistage du VIH lancé depuis 2005 au Cameroun, permet d'élargir l'accès aux prestations de dépistage à travers les campagnes de sensibilisation [5, 6]. La mobilisation importante au cours de ces activités permet de dépister un nombre considérable de personnes. Aussi, l'objet de ce travail était de dresser le profil épidémiologique de l'infection par le VIH au cours d'une campagne de sensibilisation à Yaoundé, dans l'optique de ressortir les facteurs susceptibles d'influencer sa prévalence ainsi que la perception du test de dépistage.

## Méthodes

Nous avons mené une étude transversale, descriptive et analytique qui a eu lieu pendant une semaine au cours du mois de juillet 2011 à Yaoundé au Cameroun. Nous avons obtenu le consentement éclairé de tous les participants à l'étude. Ces derniers étaient âgés de plus de 15 ans. Le dépistage de l'infection par le VIH s'effectuait à travers une unité mobile de dépistage de l'infection par le VIH [5, 6] selon l'algorithme de dépistage en série [11].

Les données collectées ont été saisies et analysées par des méthodes de statistiques descriptives et analytiques en utilisant le logiciel SPSS 19. Les différences entre les proportions ont été analysées en utilisant des tables de contingence et en appliquant le test de Chi-2. L'ampleur de l'association entre deux variables qualitatives a été évaluée par le rapport de côte pour déterminer les facteurs de risque pris individuellement (analyse univariée). Le test de Cochran-Armitage [12, 13] a été utilisé pour déterminer l'existence d'une variation linéaire de proportions en fonction d'une variable ordinale. Les valeurs p < 0,05 étaient considérées comme statistiquement significatives.

## Résultats

Un total de 911 personnes a été dépisté sur une période de 5 jours, correspondant ainsi à une moyenne de 182 personnes par jour. 24 personnes ont été dépistées séropositives, soit une prévalence de 2.6%.

### Caractéristiques sociodémographiques de la population d'étude

Le Tableau 1 représente les principales caractéristiques sociodémographiques de notre échantillon.

**Tableau 1:** Caractéristiques sociodémographiques de la population étudiée

Caractéristique	Effectif (N = 911)	Pourcentage (%)
**Sexe**		
**Femme**	446	49.0
**Homme**	465	51.0
**Ages (ans)**		
**15-24**	354	38.8
**25-34**	346	38.0
**35-44**	146	16.0
**≥45**	65	7.1
**Statut matrimonial**		
**Célibataire**	733	80.4
**Marié**	157	17.3
**Veuf/veuve(s)**	21	2.3
**Profession**		
**Elève**	58	6.4
**Etudiant**	483	53.0
**Fonctionnaire**	102	11.2
**Privé**	183	20.1
**Sans emploi**	85	9.3

La population étudiée était constituée 465 personnes de sexe masculin (51%) et 446 personnes de sexe féminin (49%). Le sexe ratio femme/homme était de 0.96. Les participants étaient âgés de 15 à 74 ans. Les personnes âgées de 15 à 24 ans étaient les plus représentées avec 38.8%. La moyenne d'âge était de 29.53 ± 10.57 ans. La population étudiée était majoritairement constituée d'étudiants (53%). Les personnes exerçant dans le secteur privé constituaient 20.1%, 11.2% des participants étaient des fonctionnaires et 9.3% n'avaient aucun emploi. Les célibataires représentaient 80.4% de notre population d'étude, les personnes mariées constituaient 17.3% et les veuf/veuves, 2.3%.

### Séroprévalence du VIH

L'association entre certaines caractéristiques sociodémographiques et la sérologie VIH est illustrée par le Tableau 2. La prévalence dans la population féminine était significativement 3 fois plus élevée que chez les hommes avec 4% contre 1.3% (p = 0.01).

**Tableau 2:** Infection à VIH/SIDA dans la population étudiée

Caractéristique	Effectif (N = 911)	Prévalence (%)	RC(2)	95%IC(2)	Valeur p du RC	Valeur p du Chi-2
**Sexe**						
**Femme**	446	18(4.0)	3.22	(1.26,8.18)	0.01	0.01
**Homme (1)**	465	6(1.3)	1.00			
**Age**						
**15-24**	354	5(1.4)	0.14	(0.04;0.48)	0,02	0.01*
**25-34**	346	10(2.9)	0.29	(0.10;0.84)	0.02	
**35-44**	146	3(2.1)	0.21	(0.05;0.85)	0.03	
**≥45(1)**	65	6(9.2)	1.00			
**Statut matrimonial**						
**Célibataire**	733	15(2.0)	0.15	(0.03;0.74)	0.02	0.02
**Marié**	157	6(3.8)	0.30	(0.05;1.68)	0.17	
**Veuf/veuve(s) (1)**	21	3(14.3)	1.00			
**Profession**						
**Elève**	58	4(6.9)	0.63	(0.18;2,1)	0.45	0.00
**Etudiant**	483	3(0.6)	0.05	(0.01;0.2)	0.00	
**Fonctionnaire**	102	1(1.0)	0.08	(0.01;0.7)	0.02	
**Privé**	183	7(3.8)	0.34	(0.12;0.9)	0.04	
**Sans emploi(1)**	85	9(10.6)	1.00			

(1) = Valeur de référence pour calcul de RC

* Valeur-p pour la tendance (trend)

(2)RC = Rapport de Cote, (2)95%IC = Intervalle de Confiance à 95%

La prévalence la plus élevée était celle retrouvée dans le groupe des personnes âgées de plus de 45 ans avec 9.2%. Elle était cinq fois plus élevée que celle des personnes âgées de 35 à 44 ans (p = 0.03), trois fois plus que la prévalence au sein des personnes âgées de 25 à 34 ans (p = 0.02). La plus faible prévalence était celle retrouvée au sein des personnes âgées de 15 à 24 ans avec 1.4%.

Les veuf/veuves avaient une prévalence de 14.3%, sept fois plus élevée que celle des célibataires avec 2% (p = 0.02). Elle était de 3.8% pour les personnes mariées. La prévalence du VIH/SIDA était plus importante pour les personnes sans emplois avec 10,6%. Elle était pratiquement vingt fois plus élevée que celle qu'on retrouvait chez les étudiants (p < 0.001).

### Sensibilisation dans la population étudiée

La participation aux campagnes de sensibilisation antérieurement ainsi que la perception de ces dernières figurent dans le Tableau 3.

**Tableau 3:** VIH/SIDA et sensibilisation dans la population étudiée

Caractéristique	Effectif n(%)	Prévalence n(%)	RC(2)	IC(2)	Valeur p du RC	Valeur p du Chi-2
**Campagne antérieure**						
Oui (1)	611(67.1)	14(2.3)	1			
Non	300(32.9)	10(3.3)	1.46	(0.46;4.7)	0.51	0.52
**Moyens de sensibilisation**						
Spots publicitaires	806(88.5)	17(2.1)	0.21	(0.06;0.75)	0.02	0.09
Publireportages	271(29.6)	9(3.3)	1.37	(0.39;4.77)	0.62	0.62
Tracts	189(20.7)	7(3.7)	0.69	(0.18;2.66)	0.59	0.59
Panneau de sensibilisation	294(32.2)	7(2.4)	0.78	(0.20;2.99)	0.72	0.72
**Qualité sensibilisation**						
Très bonne (1)	233(25.6)	4(1.7)	1			
Bonne	477(52.4)	12(2.5)	1.48	(0.47;4.63)	0.50	0.08*
Passable	178(19.5)	6(3.3)	1.99	(0.56;7.19)	0.29	
Médiocre/Nulle	23(2.5)	2(8.7)	5.45	(0.94;31.54)	0.05	

(1) = Valeur de référence pour calcul de RC

* Valeur-p pour la tendance (trend)

(2)RC = Rapport de Cote, (2)95%IC = Intervalle de Confiance à 95%

Près de 7 personnes sur 10 avaient déjà participé à une campagne de sensibilisation antérieurement contre 32.9% qui n'étaient qu'à leur première campagne de sensibilisation. La prévalence dans le premier groupe était de 2.1% contre 3.1% dans le second groupe (p = 0.51).

Une majorité des personnes interrogées (88.5%) pensaient que la sensibilisation au Cameroun est faite à travers les spots publicitaires. La prévalence était cinq fois moins importante chez ces derniers par rapport à ceux qui ne connaissait pas ce mode de sensibilisation (p = 0.02).

Selon 25.6% des personnes interrogées, la sensibilisation au Cameroun est très bonne. En effet, 52.5% la trouvaient bonne et 2.5% la jugeaient nulle ou médiocre. La prévalence de l'infection par le VIH avait tendance à augmenter selon le degré d'insatisfaction. Elle passait de 1.7% chez les personnes qui trouvaient la sensibilisation «très bonne» à 8.7% chez les personnes qui la trouvaient nulle ou médiocre. Mais cette tendance n'était que marginalement significative (p = 0.08).

### Perception du test de dépistage

La perception du test de dépistage est représentée par le Tableau 4. Plus de huit personnes sur dix (84.7%) des sujets interrogés trouvaient le test de dépistage nécessaire; 10.6% le considéraient vital et 7.9%, effrayant. La plupart des personnes dépistées n'étaient pas à leur premier test de dépistage. Seuls 23.8% des personnes dépistées l'étaient pour la première fois. La prévalence était supérieure chez ceux qui étaient à leur premier test de dépistage (3.2% contre 2.4%).

**Tableau 4:** Perception et réalisation du test de dépistage

Caractéristique	Effectif n(%)	Prévalence n (%)	RC(2)	IC(2)	Valeur p du RC	Valeur p du Chi-2
**Perception du test**						
Nécessaire	772(84.7)	18(2.3)	0.7	(0.31;1.59)	0.39	0.37
Vital	97(10.6)	4(4.1)	1.7	(0.51;5.70)	0.34	0.50
Effrayant(1)	72(7.9)	2(2.7)	1		0.99	0.96
**Test antérieur**						
Non(1)	217(23.8)	7(3.2)	1			
Oui	694(76.2)	17(2.4)	1.33	(0.47;3.87)	0.53	0.53
**Résultat retiré**						
Non	31(4.5)	3(9.7)	4.96	(1.35;18.28)	0.02	0.01
Oui(1)	663(95.5)	14(2.1)	1			
**Dépistage avec partenaire**						
Non	341(64.6)	11(3.2)	6.2	(0.79;48.4)	0.08	0.05
Oui(1)	187(35.4)	1(0.5)	1			

(1) = Valeur de référence pour calcul de RC; (2)RC = Rapport de Cote; (2)95%IC = Intervalle de Confiance à 95%

Une très grande majorité des personnes ayant déjà effectué leur test de dépistage auparavant (95.%) avaient retiré le résultat. La prévalence était cinq fois plus élevée chez celles qui n'avaient pas retiré leur résultat (p = 0.02).

La réalisation d'un test de dépistage antérieur était fonction de certains paramètres repertoriés dans le Tableau 5. En effet, les personnes de sexe féminin représentaient la plus grande proportion de celles ayant déjà réalisé le test de dépistage (80%) par rapport aux personnes de sexe masculin (p = 0.02).

**Tableau 5:** Test de dépistage antérieur suivant l'âge, le sexe et la profession

Caractéristique	Oui (n)(%)	Non (n)(%)	Valeur p du Chi-2
**Sexe**			
Femme	353(50.9)	93(42.8)	
Homme	341(49.1)	124(57.1)	0.02
**Age**			
15-24	216(31.1)	138(63.6)	
25-34	301(43.4)	45(20.7)	
35-44	128(18.4)	18(6.2)	
≥45	49(7.1)	16(7.3)	0.00
**Profession**			
Elève	33(4.8)	25(11.5)	
Etudiant	361(52.0)	122(56.2)	
Fonctionnaire	90(12.9)	12(4.5)	
Privé	133(19.2)	50(23.0)	
Sans emploi	77(11.1)	8(3.6)	0.00

Les personnes ayant déjà effectué le dépistage se répartissaient de la manière suivante en fonction de l'âge: 43.3% étaient âgées entre 25 et 34 ans, 31.1% avaient un âge compris entre 15 et 24 ans; 18.4% étaient âgées entre 35 et 44ans et 7.2% âgées de plus de 45 ans. (p < 0,001). En outre, 52% des personnes ayant déjà effectué au moins un test de dépistage auparavant étaient les étudiants, 19.2% étaient les personnes exerçant dans le secteur privé; 12.5% étaient les fonctionnaires; 11.1% les personnes sans emploi et 4.8% restant étant les élèves (p < 0,001).

### Connaissances sur le VIH/SIDA

Les réponses des participants sur la connaissance du VIH/SIDA figurent dans le Tableau 6. La totalité des sujets interrogés avaient déjà entendu parler du VIH/SIDA avec 99.1% de notre échantillon convaincus de son existence. Seuls 0.9% des personnes disaient n'en être pas convaincues et il s'agissait exclusivement de personnes séronégatives. Concernant les moyens de transmission de la maladie, les rapports sexuels ont été notifiés à hauteur de 93.2% par l'ensemble de l'échantillon; ensuite, la transfusion sanguine avec 62.5%; les objets souillés pour 54.1% et la voie maternofoetale qui a été reconnue comme moyen de transmission du VIH par 44.7%.

**Tableau 6:** Connaissances sur le VIH/SIDA

Caractéristique	Effectif	Pourcentage (%)
**Mode de contamination**		
Rapports sexuels	849	93.2
Transfusion	569	62.5
Materno-fœtale	407	44.7
Objets souillés	493	54.1
**Personnes à risque**		
Femmes	35	3.8
Hommes	9	1.0
Prostituées	77	8.5
Etudiantes	32	3.5
Camionneurs	21	2.3
Tout le monde	727	79.8
Autres	10	1.1
**Moyens de protection**		
Abstinence	370	40.6
Préservatifs	555	60.9
Coït interrompu	3	0.3
Dépistage régulier	107	11.8
Dépistage régulier avec partenaire	56	6.1

Parmi les personnes interrogées, 79.8% ont estimé que le risque de contamination par le VIH /SIDA concernaient tout le monde, contre 8.5% pour les prostituées qui sont particulièrement à risque et 3.8% pour le reste des femmes.

Les moyens de protection utilisés étaient principalement les préservatifs pour 60,9% des sujets; ensuite l'abstinence pour 40.6%. Le dépistage régulier a été mentionné par 11,8% des personnes.

### Comportement sexuel

Ces informations sont regroupées dans le Tableau 7. En effet, au cours des trois derniers mois précédant la campagne de sensibilisation, 55.6% des individus avaient eu un partenaire sexuel; 10.1% avaient eu deux partenaires sexuels et 5.2% au moins trois partenaires sexuels. Par contre 29.1% n'avaient pas de partenaires sexuels.

**Tableau 7:** Comportement sexuel

Caractéristique	Effectif	Pourcentage (%)
**Nombre de partenaires aucours des 3 derniers mois**		
0	265	29.1
1	507	55.6
2	92	10.1
3 et plus	47	5.2
**Partenaires réguliers**		
0	127	19.6
1	458	70.9
2	47	7.3
3 et plus	14	2.2
**Partenaires occasionnels**		
0	455	70.4
1	138	21.4
2	33	5.1
3 et plus	20	3.1
**Rapports non protégés avec partenaires réguliers**		
Non	208	40.1
Oui	311	59.9
**Rapports non protégés avec partenaires occasionnels**		
Non	156	81.7
Oui	35	18.3

Pour les 646 personnes qui avaient eu au moins un partenaire sexuel, 70.9% avaient un partenaire régulier, 7.3% avaient deux partenaires réguliers et 19.6% n'avaient pas de partenaires réguliers. 21.4% avaient eu un partenaire occasionnel; 5.1% et 3.1% avaient eu deux et minimum trois partenaires occasionnels respectivement.

La majorité des personnes (59.9%) qui avait au moins un partenaire sexuel régulier a eu des rapports non protégés avec celui-ci. En outre, 18.3% des individus qui avaient au moins un partenaire occasionnel déclarent avoir eu un rapport sexuel non protégé avec ce dernier. On n'a pas noté d'association significative entre le comportement sexuel des trois derniers mois et le statut sérologique.

## Discussion

Neuf cent onze personnes ont été dépistées sur une période de 5 jours, correspondant ainsi à une moyenne de 182 personnes par jour. Cette moyenne est importante bien que inférieure à celle retrouvée par Mbopi Kéou et collaborateurs, avec une moyenne de 277 personnes dépistées par jour [5, 6]. Cette importante mobilisation tend d'avantage à corroborer les conclusions d'Aguirre et collaborateurs, tirées au terme d'une campagne ayant permis de dépister 23900 personnes en deux ans aux Etats-Unis [14]. Elle serait sous-tendue par les avantages relatifs à l'anonymat, la proximité, la gratuité des tests et rapidité de disponibilité des résultats.

Les participants à cette campagne étaient à 51% des hommes. Ce chiffre est inférieur à celui retrouvé par Mbopi-Kéou et collaborateurs [6] où 64.5% des volontaires ayant participé aux campagnes de sensibilisation sur une période de trois ans étaient des hommes. Dans une société se modernisant davantage, les femmes s'assumeraient plus et seraient plus enclines à se protéger donc se faire dépister.

Les personnes âgées de 15 à 24 ans étaient les plus représentées avec 38.8%. 76.8% des participants étaient âgées de moins de 35 ans. Nkada en 2008 avait trouvé 25.6% des participants dans les campagnes de sensibilisation qui étaient âgés de 20 à 25 ans [15]. Ceci dénote ainsi d'une considération accrue de ceux-ci pour les mesures préventives contre le VIH/SIDA (dépistage en milieu universitaire avec la notion de proximité et de gratuité des tests).

La majorité de la population d'étude était constituée de célibataires et de personnes mariées avec 80.4% et 17.3% respectivement. Ces valeurs suivent la tendance observée par Koulla Shiro et collaborateurs en 1999 durant une campagne de dépistage volontaire et gratuite de cinq jours à l'Hôpital Central de Yaoundé où les groupes précédemment cités représentaient 59.4 et 29.8% [16]. Elles se rapprochent aussi de ceux retrouvées par Ndiaye et collaborateurs, en 2004 au Sénégal avec 78% de célibataires et 18% de personnes mariées [17]. Ce constat, dans notre étude, serait lié au fait que la majorité de l'échantillon était représentée par les sujets jeunes et les étudiants.

La prévalence du VIH/SIDA dans notre étude était de 2.6%. Elle est inférieure aux estimations au niveau national où elle est de 4.3% selon l'EDS-MICS 2011 [7] et de 5,3% selon l'ONUSIDA [18]. Ce fait serait lié au fait que les personnes participant aux campagnes de dépistage volontaire viennent le plus souvent «confirmer» leur séronégativité présumée et de ce fait celles subodorant leur séropositivité seraient plus réfractaire au test de dépistage. Mais pourrait-on voir dans cette faible prévalence un signe de diminution de nouvelles infections en réponse aux efforts déployés pour la sensibilisation et la prévention pour la lutte contre le VIH/SIDA.

La prévalence dans la population féminine était de 4% alors qu'elle n'était que de 1.3% chez les hommes. Cette observation suit la tendance de la féminisation de l'infection à VIH/SIDA décrite par l'EDSC III (6.8% de femmes infectées contre 4.1% d'hommes) [4], l'EDS-MICS 2011 (5.6% contre 2.9%) [7]; ainsi que par Mbopi-Kéou et collaborateurs qui avaient observés une évolution de la prévalence au sein de la population féminine allant de 6.7% en 2005 à 9.73% en 2007 [5, 6].

Dans notre étude, la prévalence augmentait avec l'âge pour se stabiliser vers 35-44 ans et remonter ensuite. Elle était plus élevée dans le groupe des personnes âgées de plus de 45 ans avec 9.2%. Elle est comparable à celle retrouvée par Nkada, en 2008 où elle était entre 7 et 8% chez les personnes âgées de plus de 45 ans [15]. Ce constat pourrait être le reflet d'une jeunesse davantage portée vers la prévention de la maladie.

La prévalence était de 2% parmi les célibataires; 3.8% parmi les personnes mariées et de 14.3% parmi les veufs/veuves. Cette dernière prévalence élevée, mais inférieure à celle retrouvée par l'EDSC III (26.4%) [4] soulève bien des questions. Les personnes ayant perdu leur conjoint adopteraient-elles des comportements à risque' Ou cette prévalence élevée serait-elle liée à une préalable exposition au virus par le conjoint le disparu'

Les personnes sans emploi représentaient le groupe avec la plus forte prévalence (10.6%). Elle est différente de celle obtenu en 2008 par Nkada [15] où elle était de 4.4%. Les élèves constituent ensuite les personnes les plus touchées avec une prévalence de 6.9%. Ce fait pourrait être en rapport avec une moindre prise de précautions de leur part. Par contre, la plus faible prévalence était celle observée au sein de la population estudiantine où elle était de 0.6%. Ainsi, les étudiants se prémuniraient plus à l'égard de l'infection compte tenu de la sensibilisation omniprésente au sein du campus, notamment à travers les panneaux de sensibilisation.

Concernant la participation aux campagnes de sensibilisation, les personnes séropositives et séronégatives avaient un taux de participation similaire à au moins une campagne de sensibilisation antérieure. Ainsi, 2/3 de la population étudiée avaient déjà participé à une campagne de sensibilisation. Ceci contraste avec la proportion retrouvée en 2008 par Nkada [15], étant de 1/3 de personnes ayant participé à une campagne de sensibilisation antérieurement. Compte tenu du fait que les unités mobiles de dépistage du VIH sont actives depuis 7 ans maintenant dans notre pays, il est donc logique qu'il y ait plus de personnes ayant déjà participé au moins une fois à leurs activités.

On constate également que la prévalence était inférieure chez les personnes qui avaient déjà participé à une campagne de sensibilisation (2,3% contre 3,3%). Ceci dénote de l'importance de la sensibilisation dans la prévention et le recul de cette infection.

Les spots publicitaires sont les moyens de sensibilisation les plus notifiés par les personnes interrogées. En outre, la prévalence retrouvée chez celles qui les ont cités était cinq fois moins importante. Ainsi, les messages véhiculés à travers ce moyen de sensibilisation, auraient un impact plus important sur le public.

Plus de 75% des personnes interrogées trouvaient la sensibilisation au Cameroun bonne ou très bonne. Le degré d'insatisfaction avait tendance à augmenter avec la prévalence. On pourrait donc dire, dans notre étude, que les séropositifs sont les moins satisfaits de la qualité de la sensibilisation. Peut-être sont-ils moins informés ou moins réceptifs aux messages de la sensibilisation.

Au sujet de la perception du test de dépistage, près de 85% des participants ont jugé le test de dépistage nécessaire; d'où la raison de leur présence à la campagne de sensibilisation. Néanmoins, 7.9% des personnes trouvent le test de dépistage effrayant. Ce pourcentage pourrait être plus important dans la population générale si le caractère effrayant du test a justement constitué une raison de dissuasion au test. Cette observation dénote une fois de plus de la pertinence de l'intensification du counselling afin de démystifier d'avantage la pathologie.

Par ailleurs, 76.2% des participants qui avaient déjà eu à effectuer un test de dépistage auparavant. Contrastant ainsi avec les 25.6% retrouvés en 2008 par Nkada [15]. On pourrait également évoquer la raison d'un nombre accru de dépistage volontaire du fait de la plus grande couverture des activités de dépistage mobiles [5, 6]. La prévalence retrouvée parmi les personnes qui n'avaient pas retiré leur résultat était cinq fois plus importante que parmi ceux qui l'avaient retiré. Les personnes ayant des comportements à risque auraient moins tendance à retirer leurs résultats par crainte d'affronter la réalité de ce résultat.

Le sexe influençait également la réalisation du test de dépistage. En effet, 80% des femmes contre 72.7% des hommes avaient déjà effectué un test auparavant. Les femmes seraient plus portées à la réalisation du dépistage et donc à la prévention. La crainte de la stigmatisation et des préjugés qui dissuadait les femmes, notamment s'agissant du dépistage dans les centres fixes [17–19] aurait moins d'influence sur leur volonté au test de dépistage. On pourrait le percevoir comme une réaction à la féminisation de l'infection à VIH/SIDA. Lorsqu'on évalue ce paramètre en fonction de l'âge, plus de sept personnes sur dix âgées qui avaient déjà effectué au moins un test de dépistage étaient âgées de moins de 35 ans. Ce qui tend à corroborer l'hypothèse de la conscientisation progressive de la jeunesse envers la prévention contre cette maladie.

En fonction de la profession, les étudiants représentent la majorité des personnes ayant déjà effectué un test de dépistage (52%). Plus de sept étudiants sur dix étaient concernés. Ce fait serait aussi lié à la sensibilisation quasi permanente au sein du campus à travers les panneaux de sensibilisation, dont ceux relatifs au dépistage. Par contre, les élèves étaient les moins bien représentés avec 4.8% (correspondant à 51.2% de l'ensemble des élèves) des personnes ayant déjà effectué un test. Serait-ce lié à une sensibilisation moins intense dans leur environnement? Ou à une moindre considération de l'importance du dépistage à leur âge?

Seules 35.4% des personnes interrogées qui affirmaient avoir déjà effectué un test de dépistage avec leur partenaire. La question du VIH/SIDA et de sa prévention ne serait pas encore pleinement abordée au sein des couples. Cette observation aurait également une répercussion sur la prévalence, étant donné que celle-ci est six fois moins importante chez les personnes qui n'avaient pas réalisé un test avec leur partenaire. Elle met en exergue le rôle positif que jouerait le dépistage en couple dans la lutte contre cette pandémie particulièrement en Afrique Subsaharienne [20].

Toutes les personnes interrogées déclaraient avoir déjà entendu parler du SIDA. 99,1% disent être convaincus de son existence. Cette situation diffère de celle du Mali où le niveau de scepticisme vis-à-vis de cette maladie serait plus important [21]. Comme moyens de contamination, les rapports sexuels ont été les plus indiqués à hauteur de 93.2% des personnes interrogées. Les autres moyens de sensibilisation étant moins cités tels que la transfusion sanguine avec 62.4%; la transmission materno-foetale avec 44.7% et à travers les objets souillés avec 54,1%. Ces résultats reflètent le fait que les messages de sensibilisation soient plus axés sur le risque de transmission par voie sexuelle qui est bien plus important que les autres.

Quant aux moyens de prévention utilisés, il s'agissait pour 60.9% des personnes de l'utilisation du préservatif. Probablement lié au fait que la majorité de la population était sexuellement active. Le dépistage régulier était pratiqué seulement par 11.7% des personnes. Bien que 76.2% des participants aient déjà effectué un test de dépistage, on se rend compte que ce dernier n'est pas vraiment encré dans les habitudes.

Concernant le comportement sexuel des 03 mois précédant la campagne de sensibilisation, le manque d'association significative entre le comportement sexuel et la sérologie VIH, suggèrerait une absence de différence entre les séropositifs et les séronégatifs concernant le nombre de partenaires sexuels réguliers ou occasionnels, l'existence de rapports sexuels non protégés avec les partenaires sexuels occasionnels et ainsi qu'au niveau des pratiques sexuelles. Ainsi, les séronégatifs ne seraient pas forcément plus prudents que les personnes séropositives. Entre les messages reçus sur la prévention et leur mise en pratique, il y aurait encore un pas à franchir [22, 23].

## Conclusion

Il apparait urgent d'intensifier les campagnes de dépistage de l'infection par le VIH en ciblant davantage des groupes particuliers tels que les élèves, les personnes âgées et les veufs/veuves, tout en recherchant les facteurs pouvant favoriser la propagation de l'infection dans ces groupes.
